# Characterization and Diversity Analysis of the Extracellular Proteases of Thermophilic *Anoxybacillus caldiproteolyticus* 1A02591 From Deep-Sea Hydrothermal Vent Sediment

**DOI:** 10.3389/fmicb.2021.643508

**Published:** 2021-03-16

**Authors:** Jun-Hui Cheng, Yan Wang, Xiao-Yu Zhang, Mei-Ling Sun, Xia Zhang, Xiao-Yan Song, Yu-Zhong Zhang, Yi Zhang, Xiu-Lan Chen

**Affiliations:** ^1^State Key Laboratory of Microbial Technology, Marine Biotechnology Research Center, Shandong University, Qingdao, China; ^2^College of Marine Life Sciences, and Frontiers Science Center for Deep Ocean Multispheres and Earth System, Ocean University of China, Qingdao, China; ^3^Laboratory for Marine Biology and Biotechnology, Qingdao National Laboratory for Marine Science and Technology, Qingdao, China; ^4^Department of Molecular Biology, Qingdao Vland Biotech Inc., Qingdao, China

**Keywords:** protease-producing bacteria, thermophilic strain, *Anoxybacillus*, extracellular proteases, deep-sea hydrothermal ecosystem

## Abstract

Protease-producing bacteria play key roles in the degradation of marine organic nitrogen. Although some deep-sea bacteria are found to produce proteases, there has been no report on protease-secreting *Anoxybacillus* from marine hydrothermal vent regions. Here, we analyzed the diversity and functions of the proteases, especially the extracellular proteases, of *Anoxybacillus caldiproteolyticus* 1A02591, a protease-secreting strain isolated from a deep-sea hydrothermal vent sediment of the East Pacific Ocean. Strain 1A02591 is a thermophilic bacterium with a strong protease-secreting ability, which displayed the maximum growth rate (0.139 h^–1^) and extracellular protease production (307.99 U/mL) at 55°C. Strain 1A02591 contains 75 putative proteases, including 65 intracellular proteases and 10 extracellular proteases according to signal peptide prediction. When strain 1A02591 was cultured with casein, 12 proteases were identified in the secretome, in which metalloproteases (6/12) and serine proteases (4/12) accounted for the majority, and a thermolysin-like protease of the M4 family was the most abundant, suggesting that strain 1A02591 mainly secreted a thermophilic metalloprotease. Correspondingly, the secreted proteases of strain 1A02591 showed the highest activity at the temperature as high as 70°C, and was inhibited 70% by metalloprotease inhibitor *o*-phenanthroline and 50% by serine protease inhibitor phenylmethylsulfonyl fluoride. The secreted proteases could degrade different proteins, suggesting the role of strain 1A02591 in organic nitrogen degradation in deep-sea hydrothermal ecosystem. These results provide the first insight into the proteases of an *Anoxybacillus* strain from deep-sea hydrothermal ecosystem, which is helpful in understanding the function of *Anoxybacillus* in the marine biogeochemical cycle.

## Introduction

Proteases, also called peptidases, account for approximately 2% of the total number of proteins in all types of organisms, which exert a variety of vital functions. Proteases are diverse, and a detailed classification of proteases is provided by the MEROPS database^1^, in which peptidases are divided into different clans and families (Barrett et al., 2001; Rawlings et al., 2008). Each peptidase is assigned to a family on the basis of statistically significant similarities in amino acid sequence, and homologous families are grouped together into a clan. Peptidases are totally divided into 7 groups based on the catalytic type, that is, aspartic peptidases (A), cysteine peptidases (C), metallo-peptidases (M), serine peptidases (S), threonine peptidases (T), mixed catalytic type (M), and unknown type (U). Each group contains several clans and has an identifier as shown in the brackets. In addition, based on their location, microbial proteases are divided into intracellular proteases, which function inside a cell, and extracellular proteases, which are secreted and function in the periplasm or outside a cell. Microbial extracellular proteases not only provide carbon and nitrogen nutrients for microorganisms, but also play a crucial role in the degradation and recycling of environmental organic nitrogen on the earth (Ran et al., 2014; Li et al., 2016).

The genus *Anoxybacillus* was first proposed in 2000, members of which are characterized by their thermophilicity, Gram-positive cell wall, and the formation of spores (Pikuta et al., 2000). So far, there are 23 species with validly published names in this genus, most of which are thermophiles isolated from terrestrial hot spring. *Anoxybacillus* strains are found to be able to secrete diverse thermophilic enzymes, including xylanase, cellulase, amylase, protease and other enzymes, which may have good industrial potentials (Bekler et al., 2016; Punam et al., 2018; Hajiabadi et al., 2019). Up to date, only a few proteases from *Anoxybacillus* have been reported. Lavrenteva et al. first reported two alkaliphilic and thermophilic *Anoxybacillus* strains secreted alkaline subtilisin-like serine proteinases (Lavrenteva et al., 2009). Later, a thermo-alkaline protease from *Anoxybacillus* sp. KP1 (Guven et al., 2015) and a thermostable alkaline protease SAPA secreted by *A. kamchatkensis* M1V (Mechri et al., 2019) were successively purified and characterized. The protease from *Anoxybacillus* sp. KP1 was stable at 50–60°C and pH 9.0 for 1 h, and was stable in the presence of detergents (Guven et al., 2015). The protease SAPA from *A. kamchatkensis* M1V was optimal at 70°C and pH 11, and showed a high detergent compatibility and an outstanding stain removal capacity (Mechri et al., 2019). In addition, Nakamichi et al. reported that the thermophilic protease secreted by *Anoxybacillus* sp. MU3 may enhance the solubilization of sewage sludge (Nakamichi et al., 2010). These studies indicate that proteases from *Anoxybacillus* may have promising potentials in industry and biotechnology.

In addition to terrestrial hot spring, thermophiles are often found in marine hydrothermal ecosystem where protease-secreting bacteria play important roles in the nitrogen cycling by decomposing dissolved and particulate organic nitrogen. Protease-secreting bacteria are often found in the diversity investigation of bacteria from hydrothermal vents. Sun et al. (2015) isolated 25 cultivable heterotrophic bacteria that exhibit extracellular protease activities from the sediments of two deep-sea hydrothermal vents of Okinawa Trough. *Vitellibacter nionensis* VBW088^T^ and *Lutibacter profundi* LP1^T^ isolated from hydrothermal vents were reported to be capable of degrading casein, indicating that they can secrete extracellular proteases (Rajasabapathy et al., 2015; Le Moine Bauer et al., 2016). In addition, based on metagenomic and metatranscriptomic sequencing on samples from deep-sea hydrothermal vent at Guaymas Basin, 59 genomes from 10 bacterial phyla contain multiple genes encoding extracellular peptidases, and 52 bacterial groups were detected to have transcripts for extracellular peptidases (Meng et al., 2016). Even so, studies on the proteolytic bacteria and their proteases from deep-sea hydrothermal vents are still quite limited. Until now, there has been no report on protease-secreting *Anoxybacillus* from marine hydrothermal vent regions.

In this study, we investigated the proteases of an *Anoxybacillus* strain, *Anoxybacillus caldiproteolyticus* 1A02591 (hereafter strain 1A02591), which was isolated from a deep-sea hydrothermal vent sediment of the East Pacific Ocean at a water depth of 2628 m. Strain 1A02591 was a thermophilic bacterium with a strong protease-secreting ability. We characterized the extracellular proteases secreted by strain 1A02591, and analyzed the diversity and functions of the extracellular and intracellular proteases of strain 1A02591 by genome sequencing and gene annotation. Moreover, the proteases secreted by strain 1A02591 cultured with casein were further identified by secretome analysis. The results showed that strain 1A02591 mainly secreted metalloproteases and serine proteases from different families, which are thermophilic and active toward different proteins, suggesting that strain 1A02591 may play an important role in the degradation and recycling of organic nitrogen in deep sea hydrothermal ecosystem.

## Materials and Methods

### Experimental Materials

*Anoxybacillus caldiproteolyticus* 1A02591 (MCCC1A02591) was obtained from Marine Culture Collection of China (MCCC), which was isolated from the deep-sea sediment collected from the hydrothermal vent in the east of the Pacific Ocean (12.71°N, 103.91°W). Casein and elastin were purchased from Sigma (United States). Insoluble type I collagen fiber (bovine achilles tendon) was purchased from Worthington Biochemical Co. (United States), gelatin from Boston Biomedical Inc. (United States), and casamino acids from Sangon Biotech Co., Ltd. (Shanghai, China).

### Analysis of the Optimum Temperature for Cell Growth and Extracellular Protease Production

To determine the optimum temperature for cell growth, strain 1A02591 was cultured at 45, 50, 55, 60, or 65°C in a liquid 2216E medium (0.5% Bacto peptone, 0.1% yeast extract and artificial seawater, pH 7.5) in 50 mL Erlenmeyer fasks, which were controlled under the atmospheric pressure and constant agitation (180 rpm). The OD_600_ of the cultures was monitored at an interval of 2 h. The growth rate of strain 1A02591 was measured by the increment of OD_600_ per hour at each temperature and was analyzed by DMfit v3.5 using Gompertz model (Baranyi and Roberts, 1994; Dey et al., 2020). To determine the optimum temperature for extracellular protease production, strain 1A02591 was cultured at 45, 50, 55, 60, or 65°C in 50 mL fermentation medium (0.3% casein, 0.2% yeast extract and artificial seawater, pH 7.5) in 500 mL Erlenmeyer fasks, which were controlled under the atmospheric pressure and constant agitation (180 rpm). The activity of the extracellular protease in the cultures was measured with casein as substrate at an interval of 24 h.

### Enzyme Assay

The activity of the protease toward casein was measured by the Folin-phenol method as previously described (Chen et al., 2003). Briefly, a reaction mixture of 100 μL enzyme in 50 mM Tris–HCl (pH 7.5) and 100 μL of 2% (w/v) casein was incubated at 70°C for 10 min. After incubation, the reaction was stopped by adding 200 μL trichloroacetic acid (0.4 M) into the mixture. Then, 100 μL of the supernatant of the reaction mixture was incubated with 500 μL of sodium carbonate solution (0.4 M) and 100 μL of the Folin-phenol reagent at 40°C for 20 min. After incubation, the OD_660_ of the mixture was measured. One unit of enzyme activity (U) is defined as the amount of enzyme that released 1 μg tyrosine from casein per min. The activities of the protease toward collagen and gelatin were assayed by the methods described by Li et al. (2016). For collagen, 5 mg collagen was incubated with 1 mL enzyme solution with continuous stirring at 70°C for 5 h. One unit of enzyme activity (U) is defined as the amount of enzyme that released 1 μmol leucine from collagen per hour. For gelatin, the mixture of 100 μL enzyme solution and 100 μL of 2% (w/v) gelatin was incubated at 70°C for 10 min. One unit of enzyme activity (U) is defined as the amount of enzyme that released 1 μmol of leucine from gelatin per hour. The activity of the protease toward elastin was determined by the photometric method (Sachar et al., 1955). The enzyme was incubated with 5 mg elastin-orcein in 50 mM Tris–HCl (pH 7.5) at 70°C for 1 h, and then the residual elastin-orcein was removed by centrifugation. The OD_590_ of the supernatant was measured. One unit of enzyme activity is defined as the amount of enzyme that released 1 nmol orcein per min.

### Characterization of the Extracellular Protease

The optimal temperature of the protease was determined by assaying the enzyme activity at pH 7.5 from 0 to 100°C. To evaluate the effect of temperature on the protease stability, the residual activity was measured at 70°C and pH 7.5 after the protease was incubated at 60, 70, or 80°C for different time intervals (15, 30, 45, 60, 75, or 90 min). The optimum pH of the protease was evaluated by assaying the enzyme activity at 70°C in the Britton-Robinson buffers at pH values ranging from 3 to 11 (Chaberek et al., 1952). The effect of NaCl concentration on enzyme activity was determined by assaying the enzyme activity at 70°C and pH 7.5 with NaCl of different concentrations from 0 to 4 M in the reaction mixture. The effects of metal ions (Li^+^, K^+^, Ca^2+^, Mg^2+^, Cu^2+^, Ni^2+^, Mn^2+^, Ba^2+^, Fe^2+^, Zn^2+^, Sr^2+^, Co^2+^ and Sn^2+^) and protease inhibitors PMSF (phenylmethylsulfonyl fluoride), EDTA (ethylene diamine tetraacetic acid), EGTA (ethylene glycol tetraacetic acid), and *o*-P (*o*-phenanthroline) on the extracellular protease were evaluated by measuring the enzyme activity at 70°C and pH 7.5 after the enzyme was pre-incubated with each metal ion or inhibitor for 1 h at 4°C. The substrate specificity of the extracellular protease was analyzed by measuring its activities toward casein, gelatin, collagen (Bovine insoluble type I collagen fiber) and elastin.

### Genome Sequencing and Analysis

The genome of strain 1A02591 was sequenced by an Illumina hiseq2000 sequencer in Shanghai Majorbio Bio-pharm Biotechnology Co., Ltd., Shanghai, China. ABySS v2.0.2 was used to do genome assembly with default parameters (with l = 15) to obtain the optimal results of the assembly (Jackman et al., 2017). GapCloser v1.12 was subsequently applied to fill up the remaining local inner gaps and to correct the single base polymorphism for the final assembly results. *Ab initio* prediction method was used to obtain gene models with Glimmer v3.02 (Delcher et al., 2007). Gene functions were annotated by Blastp using Swiss-Prot^2^, COG (Clusters of Orthologous Groups^3^) and KEGG (Kyoto Encyclopedia of Genes and Genomes^4^) (Tatusov et al., 2000; Minoru et al., 2016). tRNAs were identified using the tRNAscan-SE (v1.23^5^) and rRNAs were determined using the RNAmmer (v1.2^6^) (Lowe and Eddy, 1997; Karin et al., 2007). The obtained genome sequence was deposited in GenBank under the accession number JAEILW000000000. The signal peptides of the proteases were predicted by using SignalP^7^ (Bendtsen et al., 2004). The peptidase families were analyzed by Blastp searching against the MEROPS peptidase database Release 11.0^8^ (Rawlings et al., 2008, 2017). The amino acid sequences of peptidases were entered via the *blast merops* with a cutoff *E*-value of 1e-5.

### Secretome Analysis

Strain 1A02591 was cultured at 55°C for 12 h in the casein medium (0.3% casein, 0.2% yeast extract and artificial seawater, pH 7.5). Then, the culture was centrifuged (10,000 rpm for 20 min at 4°C) and the supernatant was collected. Proteins in the supernatant was precipitated by 50 mL acetone solution containing 10% trichloroacetic acid and 0.1% dithiothreitol overnight at −20°C. The precipitates were harvested by centrifugation for 20 min at 8,000 rpm and 4°C, washed sequentially by 80% acetone solution and 100% acetone solution, and then were dried. Subsequently, the sample was denatured by a denaturation buffer (0.5 M Tris–HCl, 2.75 mM EDTA, 6 M Guanadine-HCl), reduced by 1 M dithiothreitol, alkylated by 1 M iodoacetamide and digested by trypsin. Peptides in the sample were trapped and desalted on the enrichment column (ZipTip C18, Millipore) using 50% acetonitrile/H_2_O containing 0.1% trifluoroethanoicacid as the eluent. MS spectra of the sample were automatically recorded by the mass spectrometer Orbitrap Elite (ThermoFisher Scientific, Bremen, Germany) coupled online to an Easy-nLC 1000 (Thermo Fisher Scientific, Bremen, Germany). Finally, Thermo Scientific Proteome Discoverer^TM^ software version 1.4 was used as the research tool to analyze the secreted proteins. The secretome data was uploaded to the ProteomeXchange Consortium via the PRIDE under the accession number PXD023396.

## Results

### *A. caldiproteolyticus* 1A02591 Is a Protease-Secreting Thermophilic Bacterium

**FIGURE 1:**
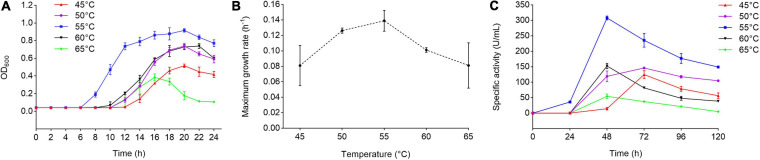
Growth and protease production of strain 1A02591 at different temperatures. **(A)** Growth curves of strain 1A02591 at temperatures from 45°C to 65°C. Strain 1A02591 was cultured in liquid 2216E medium at pH 7.5 and cell growth was monitored by measuring the OD_600_ of the culture. **(B)** Maximum specific growth rate of strain 1A02591 at 45, 50, 55, 60, and 65°C. The growth rate of strain 1A02591 was measured by the increment of OD_600_ per hour at each temperature and was fitted by DMfit v3.5 using Gompertz model. **(C)** The extracellular protease production of strain 1A02591 cultured at temperatures from 45°C to 65°C. Strain 1A02591 was cultured in a fermentation medium as described in section “MATERIALS AND METHODS,” and the protease activity in the culture was measured at 70°C with casein as the substrate. The graphs show data from triplicate experiments (mean ± SD).

Strain 1A02591 was isolated as a protease-producing bacterium from the deep-sea sediment of the hydrothermal vent in the East Pacific Ocean, and preserved in MCCC. To further characterize strain 1A02591, we analyzed the optimum temperatures for its growth and for its extracellular protease production. We speculated that strain 1A02591 was likely thermophilic based on its hydrothermal origion. Thus, strain 1A02591 was cultured at 45, 50, 55, 60, and 65°C to determine the optimum temperatures for its growth and for its extracellular protease production. When cultured in 2216E medium, the strain 1A02591 showed the best growth and the highest growth rate at 55°C (Figures 1A,B). When strain 1A02591 was cultured with casein as the carbon and nitrogen source, the extracellular protease production at 55°C was higher than those at the other temperatures, and reached the highest (307.99 U/mL) after 48 h culturation (Figure 1C). Therefore, strain 1A02591 is a protease-secreting thermophilic bacterium with an optimum temperature of 55°C for its growth and extracellular protease production.

### Characterization of the Extracellular Proteases Secreted by Strain 1A02591

**FIGURE 2:**
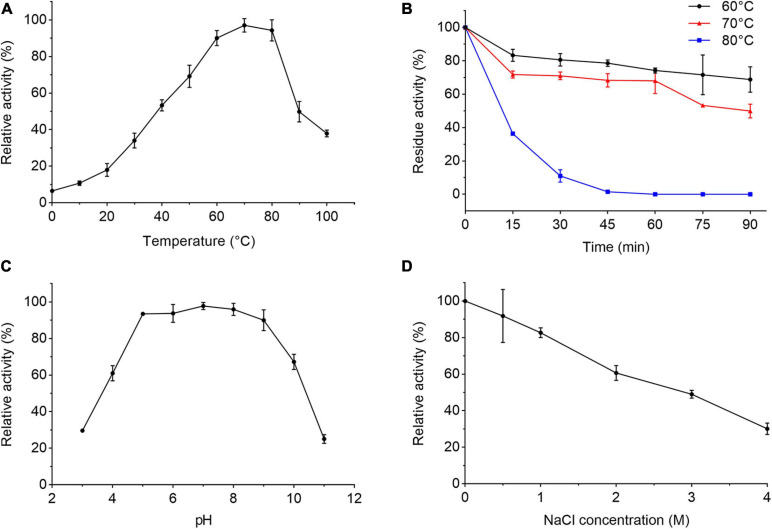
Characterization of the extracellular protease of strain 1A02591. **(A)** Effect of temperature on the protease activity. The protease activity was measured in 50 mM Tris-HCl buffer (pH 7.5) from 0°C to 100°C. **(B)** Effect of temperature on the protease stability. The residue activity of the protease was measured after the protease was incubated at 60, 70, or 80°C for different times. **(C)** Effect of pH on the protease activity. The protease activity was determined at 70°C in Britton-Robinson buffers at pH values ranging from 3 to 11. **(D)** Effect of NaCl concentration on the protease activity. The protease activity was determined at 70°C with a NaCl concentration from 0 to 4 M. The graphs show data from triplicate experiments (mean ± SD).

We further characterized the extracellular proteases secreted by strain 1A02591 cultured with casein in the medium. When the protease activity in the culture of strain 1A02591 reached the highest, the supernatant of the culture was collected and used for extracellular proteases characterization. The extracellular proteases showed the highest activity at 70°C and kept 37.9% of the highest activity at 100°C (Figure 2A), indicating that the extracellular proteases were thermophilic. The secreted proteases retained approximately 70% of its activity at 60°C and 50% at 70°C after incubation for 90 min (Figure 2B). The half-life time of the secreted proteases at 80°C was approximately 10 min. The activity of the extracellular proteases toward casein were detectable over a broad range from pH 3 to 11 with a maximal activity at pH 7 (Figure 2C). The proteases retained 89% activity in the Tris–HCl buffer containing 0.5 M NaCl, and 29% activity in the buffer containing 4 M NaCl (Figure 2D). Altogether, these results indicate that the proteases secreted by strain 1A02591 are thermophilic and neutral, with relatively good thermostability and salt-tolerance.

To determine the substrate specificity of the extracellular proteases of strain 1A02591, the activities of the extracellular proteases toward casein, gelatin, bovine insolute type I collagen fiber and elastin at 70°C were determined. As shown in Table 1, the extracellular proteases of strain 1A02591 could hydrolyze casein, gelatin and bovine insoluble type I collagen fiber, but not elastin.

**TABLE 1:** The substrate specificity of the extracellular protease of strain 1A02591.

Substrate	Activity (U/mL)
Casein	398.03 ± 8.14
Bovine insoluble type I collagen fiber	76.61 ± 7.28
Gelatin	539.24 ± 24.92
Elastin-orcein	ND

*The data represent the mean ± SD of three experimental repeats. ND means that the enzyme activity was not detectable.*

To ascertain the type of the extracellular proteases of strain 1A02591, we analyzed the effects of protease inhibitors on the protease activity by measuring the residue activity after the secreted proteases were pre-incubated with protease inhibitor for 1 h. The protease activity was almost totally inhibited by EGTA and approximately 70% protease activity was inhibited by *o*-p (a specific inhibitor of metalloproteinase) (Table 2), which indicated that metallopeptidase(s) was dominant in the extracellular proteases of strain 1A02591. In addition, 50% protease activity was inhibited by PMSF (Table 2), a specific inhibitor of serineprotease, suggesting that strain 1A02591 also secreted serine protease(s).

**TABLE 2:** Effects of metal ions and inhibitors on activity of the protease of strain 1A02591.

Metal ion (2 mM)	Relative activity*^a^* (%)	Metal ion (2 mM)	Relative activity*^a^* (%)	Inhibitors*^b^* (2 mM)	Residual activity*^c^* (%)
Control	100.00	Mn^2+^	120.22 ± 7.59	Control	100.00
Ca^2+^	99.11 ± 5.18	Ba^2+^	98.55 ± 0.17	PMSF	54.54 ± 2.55
Li^+^	104.86 ± 7.21	Fe^2+^	76.79 ± 3.41	EDTA	51.42 ± 3.59
K^+^	97.80 ± 2.05	Zn^2+^	83.20 ± 3.52	EGTA	ND
Mg^2+^	96.02 ± 1.90	Sr^2+^	102.17 ± 1.70	*o*-P	31.99 ± 2.67
Cu^2+^	72.32 ± 6.46	Co^2+^	85.87 ± 4.31		
Ni^2+^	57.14 ± 3.21	Sn^2+^	101.87 ± 1.79		

*^a^ The protease activity was measured at 70°C and pH 7.5 with casein as the substrate. The activity (298.25 ± 18.33 U/mL) of the protease without any metal ion was used as a control (100%). ^b^ PMSF, phenylmethylsulfonyl fluoride; EDTA, ethylene diamine tetraacetic acid; EGTA, ethylene glycol tetraacetic acid; o-P, o-phenanthroline. ^c^ The residual activity of the protease was measured at 70°C and pH 7.5 with casein as the substrate after the protease was incubated with an inhibitor at 4°C for 60 min. The activity (298.25 ± 18.33 U/mL) of the protease without any inhibitor was used as the control (100%). The data represent the mean ± SD of three experimental repeats. ND means that the enzyme activity was not detectable.*

The effects of metal ions on the activity of the extracellular proteases of strain 1A02591 were also investigated. Among the thirteen metal ions tested, Ni^2+^, Cu^2+^, Fe^2+^, Co^2+^, and Zn^2+^ all had an inhibitory effect on the protease activity at 2 mM, and only 2 mM Mn^2+^ increased the protease activity by 1.2 folds (Table 2).

Taken together, these results indicated that when cultured with casein in the medium, strain 1A02591 secreted thermophilic neutral proteases that may include dominant metalloprotease(s) and serine protease(s).

### Diversity and Function Analyses of the Proteases of Strain 1A02591 by Genome Sequencing and Gene Annotation

**FIGURE 3:**
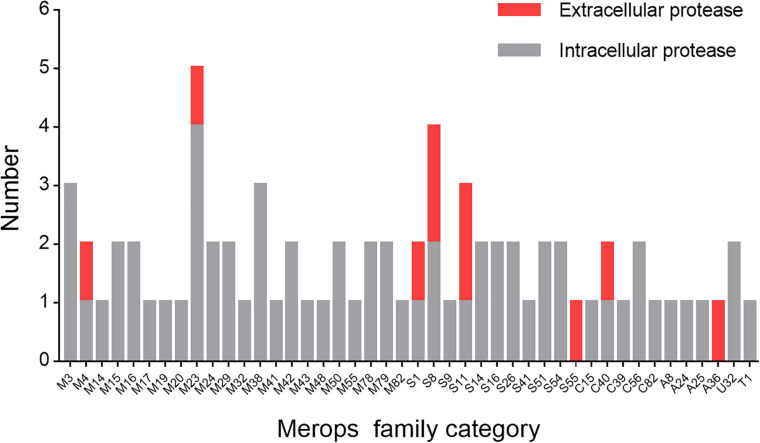
Number of the proteases of strain 1A02591 in each Merops family. The number of proteases with a predicted signal peptide is shown in red and the number of proteases without a predicted signal peptide in gray.

Next, we set out to analyze the diversity of the proteases of strain 1A02591 and their functions. First, we sequenced the genome DNA of strain 1A02591, and found out all putative proteases by gene annotation. Strain 1A02591 contains 75 proteases, which fall in 22 families of metalloprotease peptidases, 11 families of serine peptidases, 5 families of cysteine peptidases, 4 families of aspartic peptidases, 1 family of threonine peptidase and 1 family of peptidase of unknown catalytic type (Figure 3). Based on signal peptide prediction, 10 proteases have a predicted signal peptide, which are regarded as extracellular proteases. The other 65 proteases without a predicted signal peptide are regarded as intracellular proteases.

**FIGURE 4:**
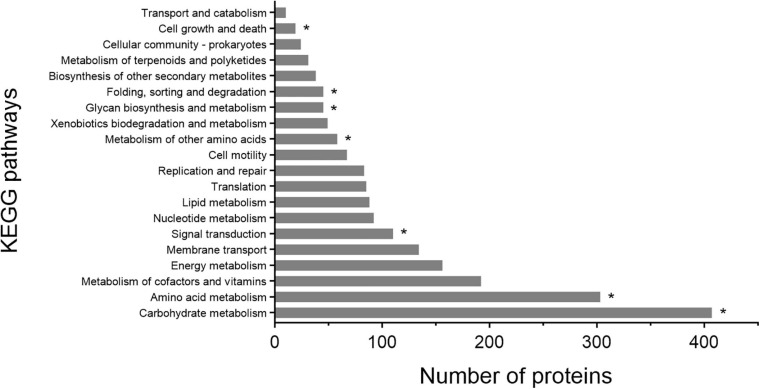
KEGG pathway classification of proteins of strain 1A02591. The asterisk represents proteases involved in a certain KEGG pathway.

Based on the KEGG pathway annotation, the metabolic pathways of strain 1A02591 were analyzed, particularly the pathways involving genes encoding proteases. KEGG analysis revealed that 2063 proteins are involved in different pathways, and the top 20 metabolic pathways according to the number of involved proteins are shown in Figure 4. Proteins involved in carbohydrate metabolism (407/2063) are the most, followed by those involved in amino acid metabolism (303/2063). It is worth noting that 10 proteases take part in 7 kinds of KEGG pathways, including carbohydrate metabolism (k01179), amino acid metabolism (k01439), metabolism of other amino acids (k01255), signal transduction (k01179), glycan biosynthesis and metabolism (K07258), folding, sorting and degradation (k03100 and k03101), and cell growth and death (k01338, k01358 and k11749).

**FIGURE 5:**
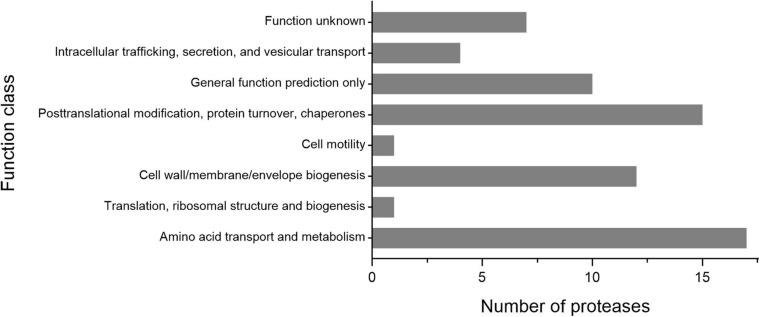
Number of the intracellular proteases of strain 1A02591 in the function classes based on COG database.

As shown in Figure 3, the 65 putative intracellular proteases of strain 1A02591 are distributed in 42 families of proteases. It has been reported that intracellular proteases play essential roles in various cell processes and pathways (Gottesman, 1996). Based on COG database, the functions of the 65 intracellular proteases of strain 1A02591 are classified into 8 categories: posttranslational modification (protein turnover, chaperones) (15/65); amino acid transport and metabolism (17/65); translation (ribosomal structure and biogenesis) (1/65); cell wall/membrane/envelope biogenesis (12/65); cell motility (1/65); intracellular trafficking, secretion and vesicular transport (4/65); general function prediction only (10/65); and unknown function (7/65) (Figure 5). Thus, a majority of intracellular proteases are involved in amino acid transport and metabolism, posttranslational modification and cell wall/membrane/envelope biogenesis in strain 1A02591. The predicted functions of these intracellular proteases are shown in Supplementary Table 1.

As shown in Figure 3, the 10 putative extracellular proteases are distributed in 8 families including 1 thermostable neutral proteinase of M4, 1 SpoIVB peptidase of S55, 1 peptidase of C40, 2 D-alanyl-D-alanine carboxypeptidases of S11, 1 sigma-E processing peptidase SpoIIGA of A36, 1 S1 peptidase, 1 M23 peptidase, and 2 S8 peptidases. Among them, the 2 proteases from families A36 and S55 are likely involved in sporulation and the development of spores (Dong and Cutting, 2004; Junne et al., 2011), the S11 proteases are likely involved in cell wall reconstruction, and the other 6 proteases from families M4, M23, S1, S8 and C40 are all likely involved in the degradation of extracellular proteins and peptides for the strain nutrition (Laskar et al., 2012; Zhao et al., 2019). Especially, the S8 proteases may have the ability to degrade collagen from animals (Ran et al., 2013), and the M23 and C40 proteases may have ability to degrade peptidoglycan from bacterial cell wall (Fukushima et al., 2018; Tang et al., 2020). The possible functions of the extracellular proteases of strain 1A02591 are detailedly shown in Table 3.

**TABLE 3:** The extracellular proteases of strain 1A02591 predicted by genome sequencing and gene annotation.

Gene ID	Family	Protease	Predicted functions
orf00069	M4	Thermostable neutral proteinase	Degrades extracellular proteins and peptides for bacterial nutrition; thermolysin-like protease
orf00545	S55	SpoIVB peptidase	Essential for the proper development of spores
orf00831	C40	Peptidase P60	Responsible for the degradation of bacterial cell wall components
orf01043 orf03925	S11	D-alanyl-D-alanine carboxypeptidase	Synthesis of bacterial cell walls, cleaving the D-Ala-D-Ala crosslinks in the cell wall peptidoglycans
orf01198	A36	Sigma-E processing peptidase SpoIIGA	Involved in sporulation
orf02124	S1	Peptidase S1	Heat shock protein that combines refolding and proteolytic activities
orf02670	M23	M23 family peptidase	Lyses cell walls of other bacteria, either as a defensive or feeding mechanism
orf03156	S8	Peptidase S8	Some function at extreme temperatures, and others at high or low pH values
orf03370	S8	Serine protease	Involved in nutrition

### Identification of the Proteases Secreted by Strain 1A02591 by Secretome Analysis

In order to ascertain the secreted proteases, strain 1A02591 was cultured with casein in the medium, and the secretome at the 12th h of cultivation was analyzed. Finally, a total of 12 proteases were found in the secretome, including 6 metalloproteases (distributed in M3, M4, M20, M23, and M32), 4 serine proteases (distributed in S8, S11, and S41), 1 cysteine protease of C40 and 1 mixed type protease of C40 (Table 4). Among them, metalloproteases were the most in both number (6/12) and abundance (62.58%), followed by serine proteases with a total abundance of 29.56%. These data were consistent with the above inhibitor experiment result that indicated that the secreted proteases of strain 1A02591 contained dominant metalloproteases and serine proteases (Table 2). Among the secreted proteases, the M4 protease was the most abundant, which has 99.82% sequence identity with thermolysin, the prototype of the M4 family, suggesting that this thermolysin-like protease secreted by strain 1A02591 is likely thermophilic, because thermolysin is a known thermophilic protease. This is consistent with the above biochemical result that the secreted proteases of strain 1A02591 were thermophilic (Figure 2A). Because proteases from families M4 and S8 all have caseinolytic activity and gelatinolytic activity (Li et al., 2016; Wang et al., 2020), the secreted proteases from these families likely contributed to the significant caseinolytic activity and gelatinolytic activity in the culture of strain 1A02591 as shown in Table 1.

**TABLE 4:** The extracellular proteases secreted by strain 1A02591 identified by secretome analysis.

Gene ID	Family	Protease	PSMs	Abundance*^a^*	Biological functions
orf00069	M4	Thermostable neutral proteinase	50	15.72%	Degrades extracellular proteins and peptides for bacterial nutrition; thermolysin-like protease
orf04095	S8	Serine protease	46	14.47%	Thermostable peptidase
orf02484	M3	M3 family	46	14.47%	Degradation of oligopeptides
orf02323	M3	Oligoendopeptidase F	37	11.64%	Degradation of oligopeptides
orf03925	S11	D-alanyl-D-alanine carboxypeptidase	31	9.75%	Synthesis of bacterial cell walls, cleaving the D-Ala-D-Ala crosslinks in the cell wall peptidoglycans
orf00077	M20	Dipeptidase PepV	30	9.43%	Hydrolyses the late products of protein degradation so as to complete the conversion of proteins to free amino acids
orf03187	M32	Carboxypeptidase M32	24	7.55%	Stable up to high temperatures
orf00831	C40	Peptidase P60	23	7.23%	Responsible for the degradation of bacterial cell wall components
orf02670	M23	M23 family peptidase	12	3.77%	Lyses cell walls of other bacteria, either as a defensive or feeding mechanism
orf03370	S8	Serine protease	10	3.14%	Thermostable peptidases
orf02654	S41	Peptidase S41	7	2.20%	C-terminal processing peptidase
orf03626	C40	Peptidase P60	2	0.63%	Responsible for the degradation of bacterial cell wall components

*^a^Abundance was calculated based on the proportion of the PSMs of a protease in the sum of PSMs of all proteases in the secretome.*

In addition, we noticed that among the 12 secreted proteases, only 5 have a predicted signal peptide, that is, 1 M4 protease, 1 S8 protease, 1 S11 protease, 1 C40 protease, and 1 M23 protease, which are likely secreted by signal peptide-mediated translocation. The other 7 secreted proteases without a predicted signal peptide might be secreted outside the cell via other secretion systems, such as the twin-arginine transport pathway, or a non-classical secretory system (Karched et al., 2019). As shown in Table 3, there are 10 putative proteases with a predicted signal peptide in strain 1A02591. Thus, in addition to the 5 ones identified by secretome analysis, 5 putative proteases with a predicted signal peptide were not found by secretome analysis, probably because these proteases were not expressed or their secretion amounts were too small to be detected in secretome analysis when strain 1A02591 was cultured with casein.

## Discussion

There is a unique light-independent ecosystem in deep-sea hydrothermal vents, where the biogroup mainly contains chemoautotrophic and heterotrophic prokaryotes and animals such as worms, mussels and shrimps (Edwards and Nelson, 1991; Plouviez et al., 2013; Kim et al., 2015). While chemoautotrophic prokaryotes produce organic molecules with energy by oxidizing the inorganic compounds available in the environments, heterotrophic prokaryotes are actively involved in carbon and nitrogen cycling by decomposing dissolved and particulate organic materials in the deep-sea hydrothermal ecosystem. Several studies have shown that protease-producing microorganisms are present in deep-sea hydrothermal ecosystem, suggesting that they are important players in the decomposition of dissolved and particulate organic nitrogen (Ladrat et al., 1995; Pikuta et al., 2007). Burrell et al. reported that extracellular protease activity was detected in the vent water bacterial community, indicating the presence of proteolytic bacteria in the hydrothermal vent of north New Zealand (Burrell et al., 2015). Sun et al. (2015) isolated 25 cultivable bacteria with extracellular protease activity from sediments associated with two deep-sea hydrothermal vents in Okinawa Trough, which belong to 9 different genera (*Bacillus*, *Exiguobacterium*, *Fictibacillus*, *Alteromonas*, *Brevibacterium*, *Rheinheimera*, *Marinomonas*, *Halomonas and Pseudoalteromonas*) (Sun et al., 2015). Genome sequence analysis revealed that *Caloranaerobacter* sp. TR13, isolated from a deep-sea hydrothermal vent on the East Pacific Rise, could encode 40 peptidases and 26 proteases including 2 subtilisin-like serine proteases, and another strain *Lutibacter profundi* LP1^T^, isolated from an Arctic deep-sea hydrothermal vent system, could encode 130 proteases (Zhou et al., 2015; Wissuwa et al., 2017). In addition, an M1 family aminopeptidase from *Pseudoalteromonas telluritireducens* DSM 16098, a strain isolated from deep-sea hydrothermal vents fluid, was cloned and characterized, which suggests that the extracellular aminopeptidase from *Pseudoalteromonas* executes the degradation of organic matters in deep-sea hydrothermal vent ecosystem (Zhang et al., 2018). These studies suggest that protease-secreting bacteria in hydrothermal vents should be actively involved in decomposing dissolved and particulate organic nitrogen, and thus play important roles in marine biogeochemical cycling. Despite these studies, as far as we know, there has been no report on protease-secreting *Anoxybacillus* from deep-sea hydrothermal vent ecosystem, although several *Anoxybacillus* strains from terrestrial hot spring have been reported to secrete extracellular proteases.

In this study, we investigated the bacterial strain *A. caldiproteolyticus* 1A02591 from a deep-sea hydrothermal vent sediment of the East Pacific Ocean. Further analysis showed that strain 1A02591 is a thermophilic bacterium and has a strong protease-secreting ability. The optimum temperature for its growth and extracellular protease production was 55°C. We characterized the extracellular proteases of strain 1A02591, and described the diversity and functions of the extracellular and intracellular proteases of this thermophilic strain by analysis of the genome sequencing, gene annotation and secretome analysis. The extracellular proteases of strain 1A02591 cultured with casein had the highest activity at the temperature as high as 70°C, indicating that thermophilic proteases are secreted by strain 1A02591. The extracellular proteases of strain 1A02591 could degrade different kinds of proteins including casein, gelatin and collagen. The extracellular proteases of strain 1A02591 was inhibited 70% by metalloprotease inhibitor *o*-phenanthroline and 50% by serine protease inhibitor PMSF, indicating that the extracellular proteases include both metalloprotease(s) and serine protease(s). Consistent with this, secretome analysis showed that metalloproteases from families M3, M4, M20, M23, and M32 and serine proteases from families S8, S11, and S41 accounted for the majority of the extracellular proteases. These secreted metalloproteases and serine proteases enable strain 1A02591 to actively degrade various exterior proteins, which provides carbon and nitrogen nutrients for itself and potentially other neighboring microorganisms, and promotes the carbon and nitrogen recycling in the deep-sea hydrothermal vent ecosystem. Thus, protease-secreting *Anoxybacillus* may play a role in the deep-sea biogeochemical cycle.

According to secretome analysis, the most abundant protease secreted by strain 1A02591 is a thermolysin-like protease. Thermolysin (EC 3.4.24.27) is a thermostable neutral zinc metalloproteinase produced by *Bacillus thermoproteolyticus*, which is the prototype of the M4 family (Yasukawa et al., 2007). Thermolysin contains 316 amino acid residues and requires one zinc ion for enzyme activity and four calciumions for structural stability (Latt, 1969; Feder et al., 1971; Titani et al., 1972). Thermolysin has been used in the production of the artificial sweetener aspartame (Birrane et al., 2014), and widely used as a non-specific proteinase to obtain fragments for peptide sequencing (Seidah et al., 1982). Thermolysin has also been used as an additive of liquid detergent due to its thermostability (Estell et al., 2019). A lot of thermolysin-like proteases have been reported, which all contain a similar catalytic domain as thermolysin (Jin et al., 1996; Tang et al., 2003). The thermolysin-like protease of strain 1A02591 shares 98.82% sequence similarity with thermolysin. It is most likely a thermostable protease based on the characters of the extracellular proteases secreted by strain 1A02591. The other biochemical characters of this thermolysin-like protease need further study.

Thermophilic proteases have various biotechnological and industrial applications. For instance, thermophilic proteases can be used as a protein degrader in waste treatment processes, e.g., the solubilization of sewage sludge (Nakamichi et al., 2010; Kun et al., 2018). They also can be used as the detergents in high-temperature conditions (Mechri et al., 2019; Emran et al., 2020). The results in this study indicate that *Anoxybacillus* strain 1A02591 is likely to be developed as a good producer of thermophilic proteases that may have various biotechnological applications. Especially, the extracellular proteases of strain 1A02591 has high activity on bovine insoluble type I collagen, implying that they may have a potential in preparing oligopeptides from collagen. Therefore, the application potentials of strain 1A02591 and its extracelluar thermophilic proteases deserve to be further studied.

## Data Availability Statement

The datasets presented in this study can be found in online repositories. The names of the repository/repositories and accession number(s) can be found below https://www.ncbi. nlm.nih.gov/genbank/, JAEILW000000000, http://www.proteomexchange.org/, PXD023396.

## Author Contributions

Y-ZZ and X-LC designed the research. X-LC and YZ directed the research. J-HC, YW, and X-YZ performed the experiments. M-LS and XZ helped in data analysis. J-HC wrote the manuscript. X-LC and X-YS edited the manuscript. All authors read and approved the manuscript.

## Conflict of Interest

XZ was employed by the company Qingdao Vland Biotech Inc. The remaining authors declare that the research was conducted in the absence of any commercial or financial relationships that could be construed as a potential conflict of interest.

## Footnotes

1 http://merops.sanger.ac.uk

2 http://uniprot.org

3 http://www.ncbi.nlm.nih.gov/COG

4 http://www.genome.jp/kegg/

5 http://lowelab.ucsc.edu/tRNAscan-SE

6 http://www.cbs.dtu.dk/services/RNAmmer/

7 http://www.cbs.dtu.dk/services/SignalP/

8 https://www.ebi.ac.uk/merops/
