# Supplementary material for: Characterization and Diversity Analysis of the Extracellular Proteases of Thermophilic Anoxybacillus caldiproteolyticus 1A02591 From Deep-Sea Hydrothermal Vent Sediment
Source: Front Microbiol. 2021 Mar 16;12:643508. doi: 10.3389/fmicb.2021.643508 (PMC8007923; doi:10.3389/fmicb.2021.643508)
Supplement: Supplementary file 1 [file Data_Sheet_1.docx]

**Characterization and diversity analysis of** **the extracellular proteases of** **thermophilic *Anoxybacillus caldiproteolyticus*** **1A02591 from deep-sea hydrothermal vent sediment**

Jun-Hui Cheng^1^, Yan Wang^1^, Xiao-Yu Zhang^1^, Mei-Ling Sun^2,3^, Xia Zhang^4^, Xiao-Yan Song^1,3^, Yu-Zhong Zhang^1,2,3^, Yi Zhang^1^*, Xiu-Lan Chen^1,3^*

^1^State Key Laboratory of Microbial Technology, Marine Biotechnology Research Center, Shandong University, Qingdao 266237, China;

^2^College of Marine Life Sciences, and Frontiers Science Center for Deep Ocean Multispheres and Earth System, Ocean University of China, Qingdao 266003, China.

^3^Laboratory for Marine Biology and Biotechnology, Qingdao National Laboratory for Marine Science and Technology, Qingdao 266237, China;

^4^Department of Molecular Biology, Qingdao Vland Biotech Inc., Qingdao, China

**Supplementary materials**

Table S1 The intracellular proteases of *Anoxybacillus caldiproteolyticus* 1A02591

| Function class | Gene ID | Family | Protease | Predicted [function](file:///C:\Users\Administrator\AppData\Local\youdao\dict\Application\7.1.0.0421\resultui\dict\?keyword=function)s |
| --- | --- | --- | --- | --- |
| Posttranslational modification, protein turnover, chaperones (energy-dependent proteases) | orf00256 | S16 | ATP-dependent protease LonB | Participates directly in overall proteolysis of misfolded proteins; sporulation under σ^F^control |
|  | orf01276 | T1 | ATP-dependent protease subunit HslV | Protein unfolding and translocation for degradation |
|  | orf02618 | S14 | ATP-dependent Clp endopeptidase, proteolytic subunit ClpP | Proteolysis of misfolded proteins |
|  | orf03562 | M41 | ATP-dependent zinc metalloprotease FtsH | Cell division protein FtsH |
|  | orf03912 | S14 | Serine protease (phage related-protein, ClpP family) | Protein quality control and the regulatory degradation of specific proteins |
|  | orf00257 | S16 | Endopeptidase La | Induced by heat shock |
| Posttranslational modification, protein turnover, chaperones (no known energy requirement) | orf04095 | S8 | Serine protease | Some function at extreme temperatures, and others at high or low pH values |
|  | orf01551 | S1 | Serine protease | The cellular response to extracytoplasmic stress |
|  | orf00361 | U32 | Protease | Modulation of host responses |
|  | orf00360 | U32 | Collagenase-like protease | Degrade soluble and reconstituted fibrillar type I collagen |
|  | orf01960 | M48 | Peptidase M48 | Substrate insertion, translocation, and ejection |
|  | orf01355  orf01356 | M16 | Insulinase family protein | Removes an N-terminal targeting signal |
|  | orf00713 | C15 | Pyroglutamyl-peptidase I | Responsible for catabolism |
|  | orf00406 | A25 | GPR endopeptidase | Germination of spores |
| Amino acid transport and metabolism | orf00182  orf00089 | M42 | M42 family peptidase | Hydrolyes N-terminal acylated amino acids as well as free N-terminal residue |
|  | orf01771  orf00099 | M29 | Aminopeptidase | Releases amino acid from peptides |
|  | orf03187 | M32 | Carboxypeptidase M32 | Hydrolyses amino acids with long side chains |
|  | orf02901 | M17 | Cytosol aminopeptidase | The breakdown of peptide products of proteinases |
|  | orf02484 | M3 | M3 family  peptidase | Degradation of oligopeptides |
|  | orf00720 | M55 | Peptidase M55 | Following hydrolysis of (D-Ala)_2_, the released D-Ala could be used as a metabolic fuel |
|  | orf01795  orf03874 | S51 | Peptidase E | Hydrolyse dipeptides |
|  | orf00046 | S9 | S9 family peptidase | Degradation of biologically active peptides |
|  | orf00483 | M14 | Peptidase M14 | Processes of bioactive peptides |
|  | orf00077 | M20 | Dipeptidase PepV | Hydrolyses the late products of protein degradation so as to complete the conversion of proteins to free amino acids |
|  | orf01368 | M19 | Membrane dipeptidase | Degradation of glutathione, cleaving the Cys-Gly dipeptide |
|  | orf02323  orf02821 | M3 | Oligoendopeptidase F | Intracellular degradation of oligopeptides |
|  | orf00143 | M24 | Aminopeptidase P family protein | Removal of the initiating methionine of many proteins |
| Translation, ribosomal structure and biogenesis | orf03496 | M24 | Aminopeptidase P family protein | Removal of the initiating methionine of many proteins |
| Cell wall/membrane/envelope biogenesis | orf01321 | M50 | RIP metalloprotease RseP | Intramembrane proteolysis |
|  | orf03626 | C40 | Peptidase P60 | Expressed during sporulation, responsible for the degradation of bacterial cell wall components |
|  | orf03683 | M15 | Peptidase M15 | Involved in bacterial cell wall biosynthesis and metabolism |
|  | orf01410 orf00297 orf02860 orf04078 | M23 | M23 family peptidase | Lyses cell walls of other bacteria, either as a defensive or feeding mechanism |
|  | orf01014 | S11 | D-alanyl-D-alanine carboxypeptidase | Synthesis of bacterial cell walls, cleaving the D-Ala-D-Ala crosslinks in the cell wall peptidoglycans |
|  | orf02654 | S41 | Peptidase S41 | Important for the degradation of incorrectly synthesized proteins |
|  | orf02584 | M15 | Peptidase M15 | Involved in bacterial cell wall biosynthesis and metabolism |
|  | orf01102 | C82 | L, D-transpeptidase | Cell-wall synthesis in bacteria |
|  | orf01209 | A8 | Lipoprotein signal peptidase | Removes the signal peptide from the N-terminus of the murein prolipoprotein, an essential step in the production of the bacterial cell wall |
| Cell motility | orf00283 | A24 | Prepilin peptidase | Type IV pilus formation, toxin and other enzyme secretion, gene transfer and biofilm formation |
| General function prediction only | orf02386 | C56 | Glutamine amidotransferase | Hydrolyzes small peptides to provide a nutritional source |
|  | orf00776 | C56 | Peptidase | The degradation of small peptides |
|  | orf02765 orf02766 orf02767 | M38 | Beta-aspartyl-peptidase | The release of isoaspartate residues from peptides which accumulate during the stationary phase of bacterial growth and may be toxic |
|  | orf00477  orf03328 | S54 | Rhomboid family intramembrane serine protease | Cleaves the transmembrane proteins |
|  | orf00971  orf03719 | M79 | CPBP family intramembrane metalloprotease | Plays an essential role in the epidermal growth factor receptor |
|  | orf01480 | M50 | Putative zinc metalloprotease Rip2 | Involved in the regulation of gene expression |
| Intracellular trafficking, secretion, and vesicular transport | orf01264 orf02427 | S26 | Signal peptidase I | Removes the signal peptides and facilitate secretion |
|  | orf01209 | A8 | Lipoprotein signal peptidase | Removes the signal peptide from the N-terminus of the murein prolipoprotein, an essential step in the production of the bacterial cell wall |
|  | orf00283 | A24 | Prepilin peptidase | Type IV pilus formation, toxin and other enzyme secretion, gene transfer and biofilm formation |
| Function unknown | orf04025 | S8 | Peptidase S8 | Degradation of misfolded proteins, regulation of short-lived proteins and housekeeping removal of dysfunctional proteins |
|  | orf00088 | M4 | Peptidase M4 | Degrade proteins and peptides for bacterial nutrition |
|  | orf02565 | C39 | Peptidase C39 | Bacteriocin-processing peptidase |
|  | orf00636 orf01378 | M78 | ImmA/IrrE family metallo-endopeptidase | Cleaving the ImmR protein |
|  | orf00951 | M82 | PrsW family intramembrane metalloprotease | Controls transcription initiation factors |
|  | orf02119 | M43 | Metalloprotease | Cleaves insulin-like growth factor binding-protein |
